# Construct validity and reliability of the physical activity parenting questionnaire for children (PAP-C)

**DOI:** 10.1186/s12966-021-01128-5

**Published:** 2021-05-05

**Authors:** Arto Laukkanen, Kaisa Aunola, Elisa Korhonen, Lisa M. Barnett, Arja Sääkslahti

**Affiliations:** 1 Faculty of Sport and Health Sciences, University of Jyväskylä, Jyväskylä, Finland; 2 Department of Psychology, Faculty of Education and Psychology, University of Jyväskylä, Jyväskylä, Finland; 3 Institute of Physical Activity and Nutrition, School of Health and Social Development, Faculty of Health, Deakin University, Burwood, Australia

**Keywords:** Physical activity, Children, Parenting, Parental control, Motivation, Self-determination theory, Assessment, Psychometrics

## Abstract

**Background:**

Children’s perception of parenting is hypothesised to significantly affect their physical activity (PA). This study aimed to examine construct validity, factorial invariance and reliability of a new tool: Physical Activity Parenting questionnaire for Children (PAP-C).

**Methods:**

PAP-C comprised 22 items hypothesised to cover 3 theory-guided factors of physical activity parenting (PAP)—namely, structure for activity, autonomy support and involvement. Construct validity and internal consistency of PAP-C were tested using confirmatory factor analysis (CFA) and composite reliability in a sample of Finnish first, second- and third graders (*n* = 456; mean age 8.77 ± 0.84 years, girls 51.1%). Factorial invariance of PAP-C across grade levels was investigated using sequential multigroup CFA. Intra-class correlation (ICC) coefficients of the sum factors were calculated in a sample of children who completed a 4-week PAP-C retest (*n* = 450; mean age 8.83 ± 0.87 years, girls 48.0%).

**Results:**

A first-order 3-factor model of the structure for activity, autonomy support and involvement, with 20 items (two items removed), showed an acceptable fit. The model demonstrated configural, metric, and scalar invariance across grade levels. Composite reliabilities indicated moderate-to-good internal consistency (from .74 to .87) for the factors. ICCs (from .494 to .750, *p* < .001) showed moderate to excellent test–retest stability for all grade levels.

**Conclusions:**

PAP-C can be considered to be a promising tool for investigating 7–10-year-old children’s perceptions of PAP.

**Supplementary Information:**

The online version contains supplementary material available at 10.1186/s12966-021-01128-5.

## Background

Physical activity (PA) has favourable associations with both physical and mental health of children and adolescents. For example, PA is linked with lower cardiovascular and metabolic risk markers, body mass [1, 2], enhanced bone health [3], higher motor competence [4], adaptive mental health outcomes and cognitive functioning [5]. However, the prevalence of physical inactivity is high [6, 7], which is a concern from the public health perspective as well as from the perspective of athletic development [8]. In order to affect PA, the key factors behind PA should be targeted. Parents and guardians are the primary socialising agents for children’s PA behaviour formation throughout childhood and thus represent a key factor to target.

Physical activity parenting (PAP), i.e. behavioural strategies employed by parents to socialise children into a physically active lifestyle, is a consistent correlate [9] and determinant of children’s PA [10]. The integrated model of physical activity parenting (IMPAP) [11] proposes that PAP consists of three parenting dimensions, namely responsiveness, demandingness and structure. The parental structure is described as organization of children’s social and physical environments to facilitate the development of competence. Demandingness and responsiveness reflect parent- and child-centeredness of the PAP practices, respectively. The IMPAP proposes that parents can directly influence their children’s PA as well as indirectly via child modifiable PA attributes, such as motivation for PA, perceived PA competence and enjoyment of PA. Importantly, child interpretation of PAP practices is expected to moderate the direct and indirect effects of PAP on child PA outcomes. The limited body of literature in this area indicates associations between child-reported PAP and child PA outcomes to vary according to the PA measurement. Child-reported PAP is shown to be significantly associated—and more strongly than parent-reported PAP—with child self-reported PA [12, 13]. Child-reported PAP is also reported as mediating the association between parent-reported PAP and child self-reported PA [14]. To date, child-reported PAP is found not to be associated with parent-reported [15] or objectively measured PA in children [16]. Therefore, the role of child-reported PAP is not yet clarified. One reason for that may be that various methodological issues limit the usage and predictive power of the current PAP measures used with children.

First, most of the measures used for inspecting children’s perception of PAP are translated from measures that were originally developed for parents. This is probably why these measures operationalise PAP in a rather unidimensional manner, typically considering direct parental influences, such as weekly frequencies of parental encouragement, modelling, supervision and logistic support for PA [16, 17]. However, recent work by our team provided preliminary evidence that—on the basis of interviews with 7–10-year-old children—it is possible to identify PAP practices that support or, alternatively, hamper development of autonomous (intrinsic) motivation for PA in children [18]. For instance, parental encouragement that reflected unconditional support and investing attention in the child’s thoughts and feelings was found to support children’s perceived competence and autonomous PA motivation but encouragement that reflected parental control (e.g. competition-oriented instead of development-oriented encouragement) contributed to controlled PA motivation. Furthermore, it was also found that co-participation—combined with competence-support and taking into consideration the child’s own PA interests—contributed to autonomous motivation, while co-participation that included forceful assertions and pressuring contributed to lower perceived competence and controlled motivation of physical activity. Therefore, current PAP measures may suffer from low discriminant validity in separating the practices that support from those that hamper children’s confidence and motivation to move.

Second, a widely acknowledged limitation of PAP measures, in general, is their loose theoretical basis [19, 20]. For instance, there is no theoretical basis presented for the widely used Activity Support Scale, which was originally developed for use with parents [21] and then later translated for use with adolescents [22] and children [23]. Third, little measure development and validation work has been done, especially with children under 10 years of age. This is a limitation because the PAP level decreases as children age [24]. In addition, the PAP form changes from co-participation to a more financial and logistic form of support [24] and the parental socialising role is gradually shared with peers, teachers and other important social agents [25, 26].

Overall, the current PAP measures used with children lack consideration of children’s motivational perspective, a theoretical basis and multidimensionality of the construct. Furthermore, there is also a lack of PAP measures validated for use with children under the age of 10—and yet, for this age group, parents are typically the most powerful socialising agents in the children’s lives. To address these limitations, the present study presents and describes the development, construct validity, factorial invariance and reliability of a new Physical Activity Parenting questionnaire for Children (PAP-C). The aim is to provide an age-appropriate, theory-guided and multidimensional PAP measure for use with children seven to 10 years of age.

### The present study

**Fig. 1:**
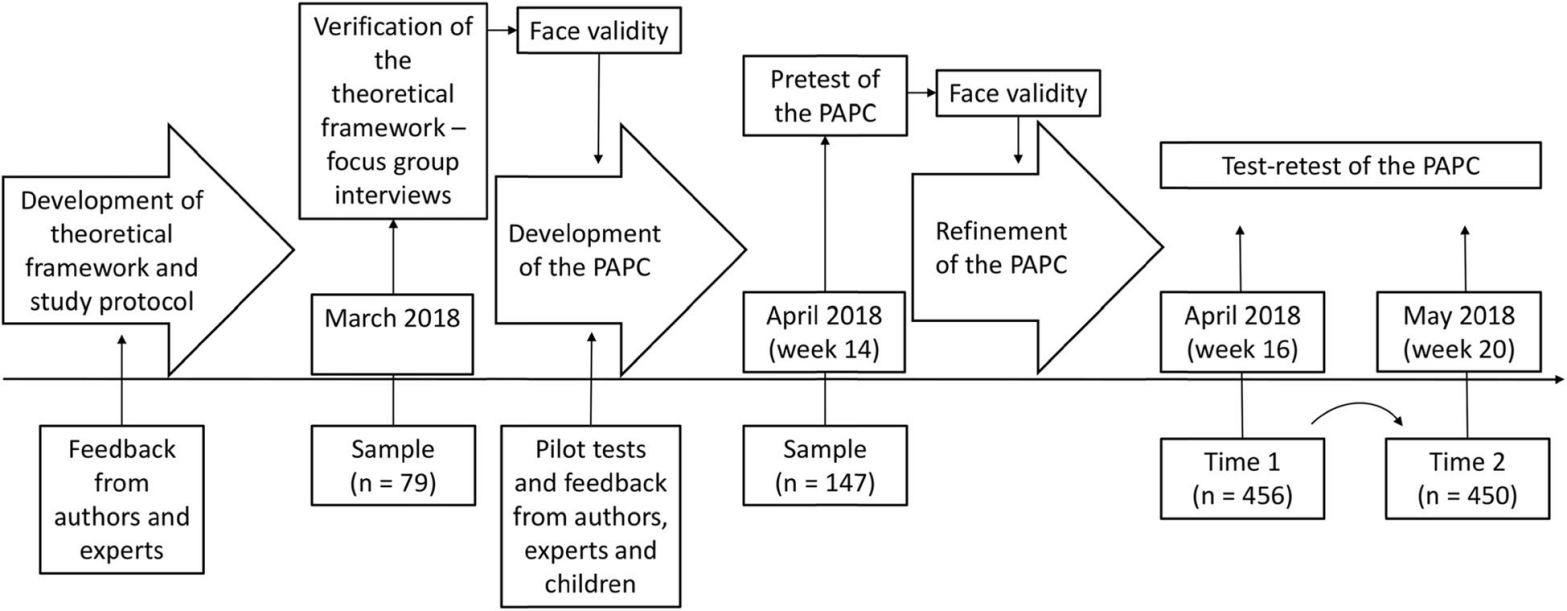
Project procedure and timeline

The PAP-C was developed for use during the Active Family study—a longitudinal study designed to investigate the bidirectional relationship between PAP and the characteristics of 7–10-year-old children (physical activity, temperament and perceived motor competence) over the transitional period from kindergarten to primary school (Fig. 1) [27]. The PAP-C aimed to examine parental influences, together with parent-reported PAP, on children’s PA and perceived motor competence—i.e. the perception of their actual movement capabilities [28].

**Fig. 2:**
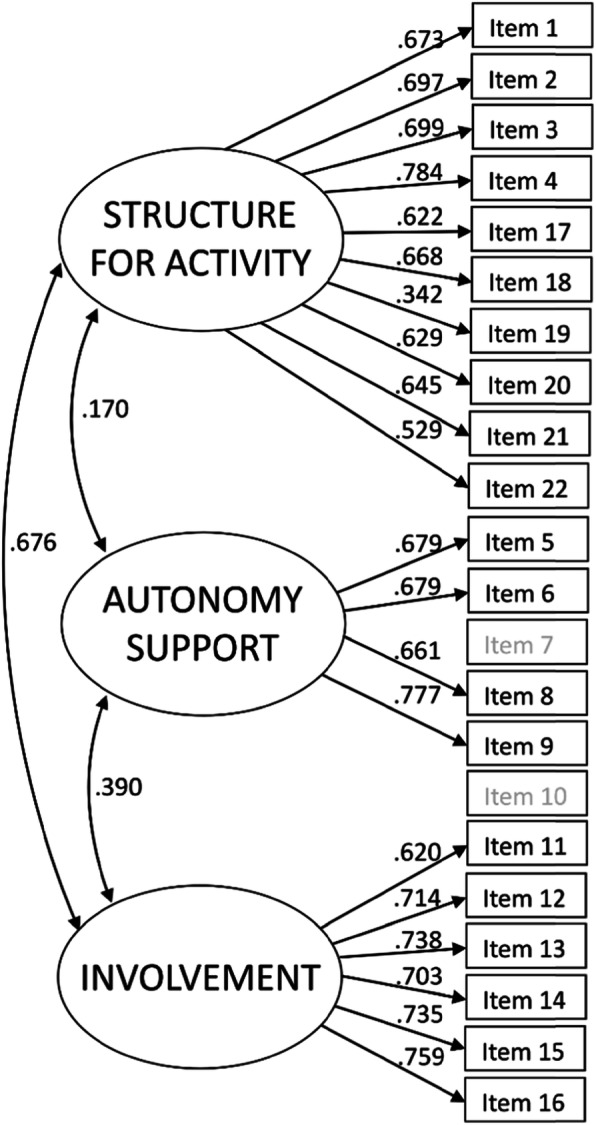
First-order 3-factor model for 20 items. Estimates are based on completely standardised solutions (Time 1)

#### Theoretical framework

A comprehensive description of the theoretical framework used in this study is previously published [18] and is briefly described here. Development and preliminary face validity testing of the PAP-C were based on focus group interviews with 7–10-year-old children (*n* = 79) [18]. The focus groups aimed to deductively provide children’s perspectives on PAP within the theoretical frameworks of parenting dimensions [29, 30] and within the key parenting dimensions that influence motivational regulation of PA according to the self-determination theory (SDT) ([31, 32], pp. 319–350). First, the parenting dimensions provided a higher-order framework for conceptualising PAP practices under two orthogonal factors: responsiveness and demandingness. Responsive parenting is characterised by warmth, supportiveness, involvement, acceptance and expressing positive feelings. Demanding parenting is characterised by limit setting, monitoring, supervision, behavioural control and knowledge of the child’s behaviour. Overall, the parenting dimensions provide insight into the manner in which parenting is provided. Current evidence shows that a PAP that is high in responsiveness and low in demandingness is favourably associated with the PA level of children [33–35]. Second, SDT provided a lower-order framework for theoretically conceptualising parental influences on children’s motivational regulation of PA. Conceptualisation of parenting practices of the PAP-C under the parenting dimensions of SDT, instead of the dimensions represented in association with the IMPAP [11], was justified by two reasons: First, the PAP-C was developed on the basis of an hypothesis that motivational self-regulation is a key mechanism through which children adopt parental influences into their PA behaviour [19]. Second, SDT provides a solid motivational theoretical mechanism which may help to understand parental influences on children’s PA behaviour formation across childhood.

Overall, SDT proposes that motivational regulation of a behaviour can be internalised as autonomous—i.e. intrinsic or externally regulated—or, alternatively, as controlled or non-regulated. Satisfaction of the basic psychological needs—namely, the needs for autonomy, competence and relatedness—is hypothesised to determine the degree to which regulation develops towards autonomous or controlled forms of motivation [31]. Furthermore, Ryan and Deci ([32], pp. 319–350) propose three key dimensions of parenting that either contribute to or hinder the satisfaction of basic needs—namely, autonomy support (i.e. taking the child’s perspective in interaction and decision-making as well as providing support and encouragement for self-expression, initiation and self-endorsed activities), involvement (i.e. the degree to which parents devote time, invest attention and resources, are caring and supportive and show warmth and concern for being actively engaged in their children’s lives) and structure i.e. the way in which parents organise children’s environment to facilitate competence—clear and consistent guidelines, expectations and rules for children as well as providing children with predictable consequences for and clear feedback about their actions). Evidence demonstrates that autonomous forms of motivation positively correlate with PA and controlled forms of motivation and amotivation negatively correlate with PA in children and adolescents [36]. In addition, need satisfaction is shown to explain significant variations in motivational regulation styles of PA for children 7–11 years of age [37].

PAP-C items were built on three SDT-derived constructs that were verified through the focus group interviews—namely, *autonomy support*, *involvement*, and structure for physical activity (hereafter, *structure for activity*). According to SDT, parental coercive control is the opposite of autonomy support. Hence, the PAP-C items that consider autonomy support were operationalised in the range of ‘autonomy supportive–controlling’ (items 5–10). On the other hand, according to SDT, the items that consider involvement and structure were operationalised in the ranges of ‘being involved–lacking attention or dedication of resources (not being involved)’ and ‘providing structure–lacking structure (not providing structure)’ (items 11–16). The items that consider parental structure were derived from previously validated questionnaires and specifically considered the structure for activity via encouragement (items 1–4) [38], modelling (items 17, 18, 20-22) [23, 39] and transportation (item 19) [22].

#### Questionnaire development

Development of the PAP-C question format was inspired by the methodologies that were designed to measure young children’s perceptions of the self—the Pictorial Scale of Perceived Competence and Social Acceptance [40]—and parent relations—the Child Puppet Interview: Parent Scales [41]. The Child Puppet Interview uses an opposing statements format that is similar to the one used by Harter and Pike [40], where a child is presented with two identical and unisex hand puppets who offer opposite statements about one of the parents and then the child is asked to choose the puppet that is more like him or her. Because the current measure was targeted for assessing groups of 7–10-year-olds at a time, the puppets were replaced by stick figures (named as ‘Tipsu’ and ‘Tapsu’) drawn on paper. The appearance of the figures was unisex and identical, except for the name label on the chest (also unisex) (Supplementary material 1). Additionally, an introductory story was used 1) to explain the concept of physical activity by describing a variety of physical activities at a light to vigorous intensity level and 2) to provide identifiable human characteristics for the figures and their families. After the introduction, the children were presented with figures representing opposite statements about each of their parents. They were asked to choose the figure that is more like him or her. Answers for each item were provided using a 4-point Likert-scale. The response scale direction was reversed for every second item to avoid response bias. Children completed the answer scale by referencing their primary female (e.g. mother, stepmother) and male (e.g. father, stepfather) guardians, who are referred to hereafter as mothers and fathers, respectively, to simplify reporting. Children living with only one guardian (male or female) reported solely on the PAP for that guardian.

The pilot-testing version of the PAP-C consisted of 22 items and was reviewed by a group of 4 researchers experienced in working with children (AL, KA, AS, DN) and a preschool teacher (SL) in order to gauge the appropriateness of the questions and identify any further questions that should be added. The feasibility and understandability of the pilot version was tested with five children who were 7–10-year-olds. A few modifications to the spelling of items were made following feedback from these children.

The PAP-C pre-test was given to 147 children in grades 1, 2 and 3 and conducted in three separate classes at each grade (mean age 8.21 ± 0.97 years; 50.0% girls). After completing the tests, the children were asked to talk about any of the items that they thought were hard to understand, irrelevant or problematic in other ways. Follow-up questions were then presented to the children (e.g. What do you understand about the question? What does the question tell you?) in order to determine face validity. Item distributions were visually inspected and strongly skewed items were modified to make distributions more uniform with the distributions of other items. For instance, the pilot item (autonomy support) ‘Parents never command Tipsu to be physically active’ versus ‘At least one of the parents commands Tapsu to be physically active’ was skewed to the lower end of the scale and rephrased in order to shift the distribution to be more normal, as follows: ‘Father/mother decides the style and the way in which Tipsu should do physical activity’ versus ‘Tapsu decides the style and way in which Tapsu is physically active’. Items that showed the lowest internal consistencies within a theoretical construct (structure α = 0.4–0.7; lack of structure α < 0.7) and had critical feedback from the children (e.g. difficulties in understanding) were removed. Internal consistencies when considering constructs of autonomy support (α = .427) and involvement (α = − 0.143; after deleting one item α = 0.458) were weak overall. Therefore, these items were rephrased. Additionally, to increase specificity, the items were further split in order to separately deal with the mother’s and father’s autonomy support and involvement. That decision was supported by SDT and previous research, both of which propose that a partner’s support significantly affects parental ability to support children’s psychological needs ([32], p. 323) and their PA [24]. The strategies used by mothers and fathers to support their children’s PA tend to differ significantly and are likely to supplement one another [24, 42]. Additionally, a child with two physically active parents is shown to be more likely to be physically active by him or herself than a child with only one active parent [43]. Consequently, information regarding the PAP of both parents separately, when available, can be hypothesised to provide the richest information regarding parental influences on children’s PA-related outcomes. For consistency, all other items on the PAP-C were also split—if they were not already—so that the mother and father could be considered separately. An exception was the item that considers the transportation of a child to PA hobbies (item 19), which was left neutral in terms of parental gender because transportation is typically conducted in turns by both mothers and fathers.

The refined PAP-C consisted of 22 items (10 items focused on parental structure for activity, six items on autonomy support and six items on involvement) (see Supplementary material 1). More structure for activity items were included than other types of support because parental structure has been identified to be highly salient [9, 44]. In the present study, construct validity of PAP-C in terms of parental structure for PA, autonomy support and involvement is investigated.

## Methods

### Sample and recruitment

The aim of the study was to examine construct validity, factorial invariance, internal consistency and the 4-week test-retest reliability of the PAP-C. It was estimated that an a priori sample size of 300–400 children would provide adequate robustness for factor analyses in three grade levels separately. Altogether, 657 informed consent forms were given to eligible children via 30 class teachers at five primary schools in three small- or mid-sized municipalities (14,000–60,000 inhabitants), located side by side in western Finland. A total of 544 (82.8%) informed consent forms with participation approval were returned. The study comprised two measurement points that were conducted within single school weeks in April and May 2018. Measurements lasted for around 25–40 min per group and were conducted in two separate groups at a time by two trained researchers (AL, SL) and with the assistance of classroom teachers in the children’s own school classes (*n* = 30) during an ordinary class hour. The first measurement point (hereafter, ‘Time 1’) comprised 456 children (Table 1), after cases with missing data (*n* = 73) were removed and cases of siblings (*n* = 15) were randomly removed. The second measurement point (hereafter, ‘Time 2’) was conducted four weeks after Time 1. Only six children could not also participate at Time 2 (Table 1).

**Table 1:** Background characteristics

Variable	Time 1^a^	Time 2^b^
N	456	450
Girls (%)	233 (51.1%)	216 (48%)
Mean age (y)	8.77 ± 0.84	8.83 ± 0.87
Grade in school		
First (%)	161 (35.3%)	160 (35.5%)
Second (%)	167 (36.6%)	163 (36.2%)
Third (%)	128 (28.1%)	127 (28.2%)
Family form^c^		
Parents live in a common household (%)	346 (75.9%)	342 (76%)
Parents live separately (%)	97 (21.3%)	96 (22.3%)
Only one parent (%)	13 (2.9%)	12 (1.7%)

^a^ Construct validity, internal consistency and factorial invariance; ^b^ Test–retest reliability. ^c^Child-reported

### Statistical analyses

The means and standard deviations of the background characteristics were calculated. The differences in characteristics were tested between boys and girls using independent sample *t*-tests (age) and chi-square (χ^2^) tests (grade, family form). The PAP-C items were investigated with confirmatory factor analysis (CFA) using the R-program (version 4.0.3) package *lavaan* [45]. A diagonally weighted least squares estimator, with a full weight matrix and a mean- and variance-adjusted test statistic (WLSMV), was used because of non- multivariate normal, ordinal items. Because some items on the questionnaire were unique for families with two parents and could not be answered by children with single parents (*n* = 13), pairwise deletion was used for missing data. All models were performed at one level (participant).

A CFA model with three latent theory-guided first-order factors, in which each item loaded on one of the factors (structure for activity, autonomy support, involvement) with freely estimating between-factor correlations was fitted for a data with all grade levels comprised. Finally, composite reliabilities—i.e. latent factor reliabilities—based on standardised factor loadings were calculated to examine the internal consistency of factors. The goodness-of-fit of the CFA models was evaluated using the comparative fit index (CFI), the Tucker-Lewis index (TLI), the standardised root-mean-square residual (SRMR) and the root-mean-square error of approximation (RMSEA). CFI and TLI values close to or above .95, RMSEA values of .06 or less and SRMR values of less than .08 were used to indicate a good fit for the models [46].

The factorial invariance of the PAP-C factor model across grade levels was investigated using sequential multigroup CFA. A multi-group CFA model without any constraints for model parameter was estimated, for testing configural invariance. Then, metric and scalar invariances were tested by setting model parameters to be equal across grade levels: loadings for metric invariance and loadings and thresholds for scalar invariance.

A 4-week test–retest reliability of PAP-C items and factors (i.e. sum of items for structure for activity, sum of items for autonomy support, and sum of items for involvement) was tested using intra-class correlation (ICC) coefficients. A two-way random effect ICC with absolute agreement type was used [47]. The ICCs were interpreted as: 0.21–0.40 fair agreement, 0.41–0.60 moderate agreement, 0.61–0.80 substantial agreement and 0.81–1.00 excellent agreement [48]. The level of significance was set at *p* < .05 in all analyses.

## Results

Background characteristics for study participants are shown in Table 1. No significant gender differences were found for any age, grade or family form between boys and girls.

### Construct validity

First, the construct validity of PAP-C was evaluated using CFA. The fit of the tested models including 22 items and factors for the structure for activity, autonomy support and involvement was: χ2 (206) = 437.821, CFI = 0.945, TLI = 0. 938, RMSEA = 0.050, SRMR = 0.064. Inspection of modification indices suggested that one item (i.e., item 7 “Dad gives Tipsu strong advice and often does so while Tipsu is physically active (says things like: ‘Go hard, hard!’, ‘Focus!’, ‘Not like that!’ versus Dad rarely gives Tapsu strong advice while Tapsu is physically active”) showed large residual correlations with various other variables, and, therefore, excluding the item from the model would improve the fit of the model. Consequently, this item, as well as its paired variable—a question equivalent for mother (item 10)—were removed from the model to ensure a structure in which both parents’ emphasis is equal in the model. After this specification, the model fitted the data better (χ2 (167) = 359.046, CFI = 0.949, TLI = 0.942, RMSEA = 0.055, SRMR = 0.061) (Fig. 2). The fit indices of the 20-item model when tested separately for each grade level are presented in Supplementary material 2. Completely standardised factor loadings for the final 20-item model were fairly high (presented in Table 2). Composite reliabilities were 0.74 for autonomy support (4 items), 0.86 for involvement and 0.87 for structure for activity (Table 3). Inter-factor correlations were moderate between structure for activity and involvement, and weak between autonomy support and support for activity and autonomy support and involvement (Table 3).

**Table 2:** Completely standardised factor loadings

Item	Time 1		
Structure for activity	Loading	*z*	SE
1. Mum doesn’t always encourage Tipsu to do physical activity or sports versus Mum always encourages Tapsu to do physical activity or sports.	0.673	20.44	0.033
2. Mum is always willing to help Tipsu in every way when it comes to physical activity and sports versus Mum isn’t always willing to help Tapsu in every way when it comes to physical activity and sports. (Reverse code)	0.697	21.31	0.033
3. Dad doesn’t always encourage Tipsu to do physical activity or sports versus Dad always encourages Tapsu to do physical activity or sports.	0.699	21.82	0.032
4. Dad is always willing to help Tipsu in every way when it comes to physical activity and sports versus Dad isn’t always willing to help Tapsu in every way when it comes to physical activity and sports. (Reverse code)	0.784	28.02	0.028
17. Tipsu thinks dad is physically active often (for example, dad goes for walks, goes to the gym, or plays ball games) versus Tapsu thinks dad is not physically active often. (Reverse code)	0.622	17.09	0.036
18. Tipsu isn’t often physically active with mum and dad versus Tapsu is often physically active with mum and dad.	0.668	20.02	0.033
19. Parents often drive Tipsu to physical activities or sports practice versus Parents don’t often drive Tapsu to physical activities or sports practice. (Reverse code)	0.342	6.27	0.053
20. Tipsu is not physically active with mum versus Tapsu is physically active with mum (for example, walks, cycles or does sports).	0.629	17.21	0.037
21. Tipsu is physically active with dad (for example, walks, cycles or does sports) versus Tapsu is not physically active with dad. (Reverse code)	0.645	18.44	0.035
22. Tipsu thinks mum is not physically active often versus Tapsu thinks mum is often physically active (for example, mum goes for walks, goes to the gym, or does sports).	0.529	12.48	0.042
Autonomy support			
5. Dad often decides the style and way that Tipsu should do physical activity (for example, he might say: ‘Not like that, like this!’) versus Tapsu decides the style and way that Tapsu is physically active.	0.679	16.17	0.042
6. Tipsu gets to decide how long Tipsu will be physically active for versus Dad decides how long Tapsu has to do physical activity for (says things like: ‘Keep going, don’t stop!’). (Reverse code)	0.679	16.17	0.042
7. Dad gives Tipsu strong advice and often does so while Tipsu is physically active (says things like: ‘Go hard, hard!’, ‘Focus!’, ‘Not like that!’) versus Dad rarely gives Tapsu strong advice while Tapsu is physically active. ^a^			
8. Tipsu decides the style and way that Tipsu is physically active versus Mum often decides the style and way that Tapsu should do physical activity (for example, he might say: ‘Not like that, like this!’). (Reverse code)	0.661	15.41	0.043
9. Mum decides how long Tipsu has to do physical activity for (says things like: ‘Keep going, don’t stop!’) versus Tapsu gets to decide how long Tipsu will be physically active for.	0.777	20.03	0.039
10. Mum rarely gives Tipsu strong advice while Tipsu is physically active versus Mum gives Tapsu strong advice and often does so while Tapsu is physically active (says things like: ‘Go hard, hard!’, ‘Focus!’, ‘Not like that!’). (Reverse code) ^a^			
Involvement			
11. Dad sometimes ignores it if Tipsu gets tired while being physically active versus Dad always notices it if Tapsu gets tired while being physically active (asks, for example, ‘Can you keep going?’).	0.620	16.63	0.037
12. Dad always listens carefully to what Tipsu has to say about being physically active versus Dad doesn’t always listen carefully to what Tapsu has to say about being physically active. (Reverse code)	0.714	22.06	0.032
13. Dad doesn’t always consider what kind of physical activity Tipsu would or wouldn’t like to do versus Dad always considers what kind of physical activity Tapsu would or wouldn’t like to do.	0.738	24.94	0.030
14. Mum always notices it if Tipsu gets tired while being physically active (asks, for example, ‘Can you keep going?’) vs. Mum sometimes ignores it if Tipsu gets tired while being physically active. (Reverse code)	0.703	21.31	0.033
15. Mum doesn’t always listen carefully to what Tipsu has to say about being physically active versus Mum always listens carefully to what Tapsu has to say about being physically active.	0.735	21.46	0.034
16. Mum always considers what kind of physical activity Tipsu would or wouldn’t like to do versus Mum doesn’t always consider what kind of physical activity Tapsu would or wouldn’t like to do. (Reverse code)	0.759	25.09	0.030

^a^Fit statistics (Loading, *z* and SE) for the removed items [7] and [10] were in the 22 items model as follows: 0.604, 13.649, 0.044 and 0.604, 13.649, 0.044, respectively

*Notes on administering the questionnaire*: Items are numbered to indicate presentation order in the PAP-C. The factor headings (Structure for activity, Autonomy support, Involvement) should not be used when administering the questionnaire

Response anchors (put a cross on the box underneath Tipsu / Tapsu): 1–Just like me, 2–A bit like me, 3–A bit like me, 4–Just like me

Instruction to child: If you haven’t got a mum or dad, put a cross on the symbol in the upper left corner of the item that considers this parent and leave the box underneath Tipsu and Tapsu empty

**Table 3:** Factor means, SD, ranges, reliabilities and intercorrelations for all grade levels together

					Scale correlations^b^			
Factor	Mean	SD	Range	α^a^	Factor (final model with 20 items)	1	2	3
1. Structure for activity	3.09	0.61	1–4	.87	1.	–	.17	.68
2. Autonomy support					2.		–	.39
6-items	3.17	0.63	1–4	.78				
4-items	3.18	0.71	1–4	.74				
3. Involvement	3.12	0.71	1–4	.86	3.			–

^a^ Composite reliability; ^b^ Inter-factor correlations

### Factorial invariance

Next, the measurement invariance of the PAPC factor model across grade levels was tested. The results are shown in Table 4. The results (Table 4) showed that when testing (a) configural invariance, (b) metric invariance, and (c) scalar invariance of the model across grade levels, the fit of the model remained adequate and adding constraints to the model did not worsen the fit, suggesting invariance of the factor structure of the PAP-C across class levels.

**Table 4:** Factorial invariance of the first-order 3 factor model across grade levels

Model	Factorial invariance	χ2	df	∆χ2	∆df	CFI	TLI	RMSEA	SRMR
1	Configural	805.638	501			0.927	0.917	0.064	0.093
2	Metric^a^	814.524	535	38.354	34	0.934	0.930	0.058	0.098
3	Scalar^b^	879.415	609	100.18	108	0.936	0.940	0.054	0.094

^a^The model is compared to model 1. ^b^The model is compared to model 2

### Test–retest reliability

The test–retest stability of the sum scores for the structure for activity, autonomy support (4 items), and involvement were moderate to substantial for the whole data (Table 5). When tested separately for different grade levels, the test-retest stabilities were moderate to substantial for the first and second graders and substantial to excellent for the third graders (Table 5). ICCs for all items included in the structure for activity varied between 0.404 and 0.680 in the whole data suggesting moderate to substantial test–retest reliability at the item level. ICCs for all items of autonomy support and involvement were between 0.345 and 0.408 and between 0.295 and 0.401, respectively, suggesting fair to moderate reliability. The items removed from the final model of CFA (items 7 and 10) showed the lowest stability out of the autonomy support items in the whole data. In general, ICCs showed an increasing trend together with age. Of all 22 items, first and second graders showed at least moderate reliability for 10 to 12 items, which was the case for all except one item for the third graders (item 13, ICC < .400).

**Table 5:** A 4-week test–retest reliability (Time 1-Time 2)

Item	First graders	Second graders	Third graders	All
Sum of items for Structure for activity	.705	.782	.750	.759
1	.341	.382	.478	.404
2	.429	.529	.589	.517
3	.383	.456	.735	.506
4	.507	.493	.737	.571
17	.663	.618	.756	.680
18	.457	.528	.586	.528
19	.550	.567	.482	.537
20	.465	.580	.600	.545
21	.498	.495	.672	.550
22	.576	.577	.583	.588
Sum of items for Autonomy support^a^	.493	.553	.602	.546
5	.411	.383	.529	.436
6	.284	.383	.472	.371
7	.304	.254	.517	.355
8	.423	.292	.425	.387
9	.259	.446	.498	.408
10	.221	.429	.405	.345
Sum of items for Involvement	.538	.510	.680	.584
11	.325	.334	.549	.401
12	.340	.299	.524	.388
13	.361	.291	.338	.340
14	.337	.251	.515	.366
15	.264	.162	.485	.295
16	.230	.322	.498	.336

^a^Items 7 and 10 were excluded from the sum

## Discussion

This research study is part of a process that aims to develop a theory-guided PAP measure for young children. Previous study by our research team provided information about 7–10-year-old children’s perceptions of PAP in terms of their responsiveness and demandingness [29, 30] and in terms of the support they provide for regulation of physical activity motivation ([31, 32], pp. 319–350). At the same time, findings provided preliminary face validity for PAP-C constructs [18]. The results of the current study provide evidence that 7–10-year-old children’s perceptions of PAP can be measured using the PAP-C with reasonable stability. Consistent with SDT’s theoretical conception regarding parenting ([32], pp. 319–350), the three parenting components of PAP-C—namely, the structure for activity, autonomy support and involvement—appeared to be statistically distinct constructs. Overall, the findings support PAP-C as a child-report measure of PAP, and this is the first measure of PAP constructed theoretically on the children’s motivational self-regulation perspective.

Generally, the test–retest reliability of children’s PAP estimates is a largely unknown area. The 4-week test–retest reliabilities found for the third graders for the structure for activity are comparable or somewhat lower in comparison to the reliabilities found for 10–11-year-old children elsewhere using similar construct types [39]. The 1-week ICCs, reported by Jago et al. [39], were between 0.60 and 0.80 for constructs that consider parental structure for PA—e.g. provision of direct support for PA, rules for PA and parental own PA. It should be noted the interval between reliability tests in the present study was a bit longer (4 weeks vs. 1 week) compared to Jago et al. (2009), a fact which may partly explain the lower stability estimates with PAP-C. However, the constructs of autonomy support and involvement—which are conceptualised in the present study—are new in the PAP field and there are no comparable measures designed for children to date. The test–retest reliabilities were lower in these constructs overall, reflecting that it was challenging for children to provide reliable estimates of these more abstract PAP constructs. This was especially the case with the first and second graders (7–9-year-olds), who showed moderate reliabilities—i.e. ICCs between .49 and .53 for the sum of items for autonomy support and involvement. It is likely the perceptions of younger children are more situational and that recent happenings in family life can influence their perceptions of parenting more than in older children, whose perceptions could be more established. However, the lower reliabilities may also relate to the quality of the items themselves and to the method of assessment because even children 5 to 6 years of age have been found to provide reliable estimates of parental structure, warmth/responsiveness and hostility [41]. To obtain this finding, Sessa et al. [41] used an individual puppet interview method, which differs from the combined text and figures method and small group data collection method used in the present study. Reid et al. [49] found that 6–12-year-olds can provide stable social support estimates when using a collaborative, interactive dialogue method. However, they also found that the children reported significant variations for social support when undergoing major family upheaval. In the present study, information obtained about the family was limited to the family form (core family, separated, single parent), which was not found to be associated with stability estimates (data not reported here). Therefore, more developmental work is needed to enhance stability, especially of the PAP-C constructs of autonomy support and involvement.

Special attention was paid to develop the PAP-C so that it fits even the youngest participants of the study, namely 7-year-olds. Tests of factorial invariance across the grade levels confirmed that the PAP-C is valid for the aimed age range (7–10-years). This is an important note as research indicates that PAP differs in both quantity and quality for children of different ages [18, 24, 26]. Practically taken it means that the needs for parental support changes along the child’s development and also the needs for the measure of PAP along with that. In previous validation studies the PAP measure psychometrics have been examined for varying age ranges—namely, for 5–8-year-olds, 9–12-year-olds [23], 9–10-year-olds [38], 10–11-year-olds [39] and 12–18-year-olds [15]. Overall, it is recommended for the validity of the PAP measures to be carefully considered for children of different ages and separately for each sample.

The construct of autonomy support was found challenging to quantify. Autonomy support is a central parenting construct, according to the SDT ([32], pp. 319–350), and it is shown to be associated with motivational regulation of PA in children [50]. Based on focus group interviews with 7–10-year-olds, it was identified that the need for autonomy is satisfied through parental support, trust for self-determined PA, unconditional approval and encouragement for PA as well as support for quitting or changing a hobby [18]. However, several challenges exist when quantifying these issues in a reliable and valid manner for children. First, the developmental phases of children steer the degree of autonomously regulated behaviours in general. Thus, parents adjust the messages they use to match their children’s developmental stages [51]; hence, the children’s perceptions of PAP also differ according to their developmental phase. Second, family situations are heterogeneous in terms of physical and social living circumstances (location, walkability/security of neighbourhood, affordances for self-determined PA, family form, etc.) and it is also likely that they moderate the children’s opportunities for self-determined PA. For instance, a parent’s perception of neighbourhood safety is shown to be associated—both cross-sectionally and longitudinally—with the time children spend being physically active and sedentary [52]. The items of autonomy support should, therefore, be neutral for a child’s developmental phase and environmental circumstances. For instance, the statements reflecting strong and directive provision of instructions used in the present study (items 7 and 10) showed an acceptable internal consistency with the other autonomy support items for second and third graders but not for first graders—a finding that likely reflects the fact that parenting differs for these items between age groups. When validating the PAP-C in the future, the testable items could attempt to quantify the items from the parent PAP reports [44] in order to reflect parental encouragement in combination with rationale and reasoning provisions, including encouragements such as ‘Parent tries to encourage child to do physical activities by telling s/he will make new friends’ or praises such as ‘Parent tells child s/he is doing well in physical activities or sports’. These types of statements could reflect the provision of autonomy and supportive encouragement, without being confounded by developmental issues or environmental biases. Overall, more qualitative work is needed, along with quantitative measure development, in order to identify a reliable way of quantifying parental autonomy support for young children.

The strengths of the PAP-C development and validation presented in this paper relate to the theory guidance, multidimensionality and appropriateness for the whole age range of 7–10-years. The PAP-C development was informed by recent qualitative work and its items assess the issues that were found to be relevant under the theoretical frameworks of parenting [18]. Thus, the items could be considered to have face and content validity. Special attention was paid to developing an age-appropriate and developmentally suitable tool. The items included for the involvement and autonomy support concepts address new constructs that have not been reported among children before—which also means that there is no study yet against which these items could be compared. Evidence of the potential utility of the PAP-C is needed in the future. On the other hand, a limitation of this study lies in its lack of motivational measures, such as measures of self-determination, perceived competence or enjoyment of PA even though a key tenet of the PAP-C supposes that parental influences occur in children’s PA through motivational processes [18]. This issue needs more attention in future studies. Another limitation relates to the limited background information of study participants, which resulted from the aspiration to optimise sample representativeness by not using parental questionnaires.

## Conclusions

The PAP-C that assesses the parental structure, autonomy support and involvement for PA was shown to be reliable and internally consistent for children seven to 10 years of age. Additionally, the PAP-C was shown to be invariant across the grade levels. The PAP-C is the first measure theoretically designed to capture child-reported parental influences on both children’s PA and their motivation for PA. It may contribute to explaining the role of child’s perceptions of PAP in children’s PA behaviour and motivation for PA.

## Supplementary Information

**Additional file 1.** Physical activity parenting questionnaire for children.

**Additional file 2.** Summary of the fit statistics for structure for activity, autonomy support and involvement for the grade levels separately.

## Data Availability

The datasets that were used and/or analysed during the current studies are available from the corresponding author upon reasonable request.

## Abbreviations

PA: Physical Activity
PAP: Physical Activity Parenting
PAP-C: Physical Activity Parenting questionnaire for Children
IMPAP: Integrated Model of Physical Activity Parenting
SDT: Self-Determination Theory
CFA: Confirmatory Factor Analyses
CFI: Comparative Fit Index
TLI: Tucker-Lewis Index
RMSEA: Root Mean Square Error of Approximation
SRMR: Standardised Root-Mean-Square Residual
ICC: Intra-Class Correlation
