# Supplementary material for: Construct validity and reliability of the physical activity parenting questionnaire for children (PAP-C)
Source: Int J Behav Nutr Phys Act. 2021 May 5;18:61. doi: 10.1186/s12966-021-01128-5 (PMC8097989; doi:10.1186/s12966-021-01128-5)
Supplement: Supplementary file 1 — Additional file 1. Physical activity parenting questionnaire for children. [file 12966_2021_1128_MOESM1_ESM.docx]

Supplementary material 1

*Physical activity parenting questionnaire for children*

**Read aloud to the children**

“I will tell you about two children, about their physical activity, and about their families. These children have sometimes tried all kinds of physical activity, such as going outdoors, cycling, running, walking their dogs, playing ball games, doing parkour, and doing aerobics. Sometimes they get out of breath when they do physical activity, and other times they don’t. For example, they don’t usually get out of breath when playing on swings, but when they run around a lot, they can get very out of breath.”

“These two children are called Tipsu and Tapsu (point to the picture). Their families are a bit different, but the children are good friends. Think about which one of them is most like you. Let’s practise using the picture below. Tipsu bakes with mum (left), Tapsu does not bakes with mum (right). Which one of them is most like you? Only choose one of the children by putting a cross on their picture.”

“If you chose Tipsu, think about whether Tipsu is just like you, or only a bit like you. Choose one of the options by checking the large or small box underneath Tipsu. If you chose Tapsu, think about whether Tapsu is a bit like you, or just like you. Choose one of the options by checking the small or large box underneath Tapsu. The “Just like me” box is always bigger than “A bit like me” box. If you don’t have a mum or a dad, you can put a cross at the symbol next to the question (a bun, flower, tree, etc.). Let’s continue in the same way. Turn over the page.”

|  |  |  | |  |  |  | |  |
| --- | --- | --- | --- | --- | --- | --- | --- | --- |
|  | 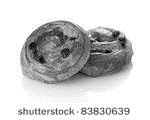 | Tipsu bakes with mum | |  |  | Tapsu does not bake with mum | |  |
|  |  | *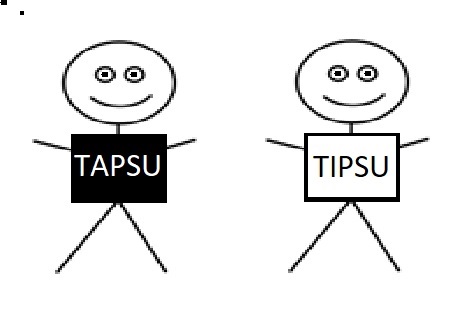* | |  |  | *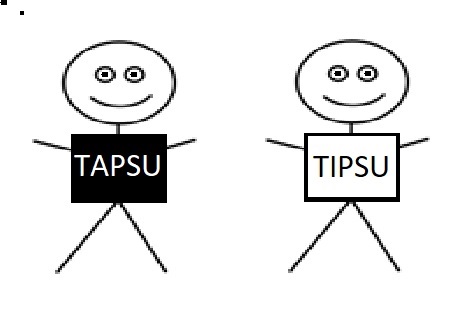* | |  |
|  |  | **□** | **□o** |  |  | **□o** | **□** |  |
|  |  | Just like me | A bit like me |  |  | A bit like me | Just like me |  |
|  |  |  |  |  |  |  |  |  |

|  |  |  | | 1 |  |  | |  |
| --- | --- | --- | --- | --- | --- | --- | --- | --- |
|  | 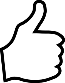 | Mum doesn’t always encourage Tipsu to do physical activity or sports | |  |  | Mum always encourages Tapsu to do physical activity or sports | |  |
|  |  | *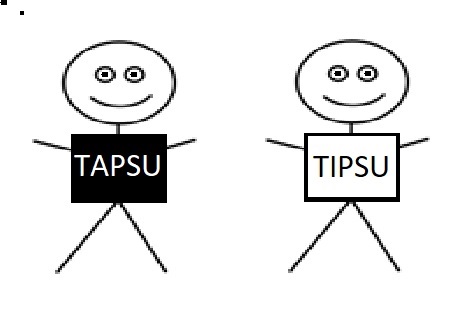* | |  |  | *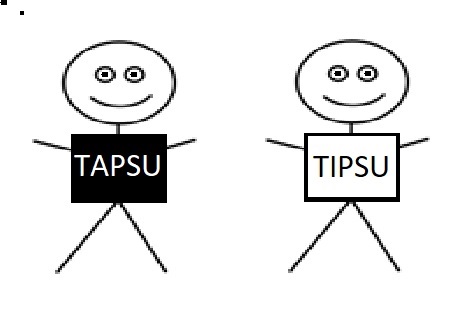* | |  |
|  |  | **□** | **□o** |  |  | **□o** | **□** |  |
|  |  | Just like me | A bit like me |  |  | A bit like me | Just like me |  |
|  |  |  |  |  |  |  |  |  |

|  |  |  | | 2 | |  | |  |
| --- | --- | --- | --- | --- | --- | --- | --- | --- |
|  | 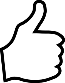  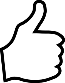 | Mum is always willing to help Tipsu in every way when it comes to physical activity and sports | |  |  | Mum isn’t always willing to help Tapsu in every way when it comes to physical activity and sports | |  |
|  |  | *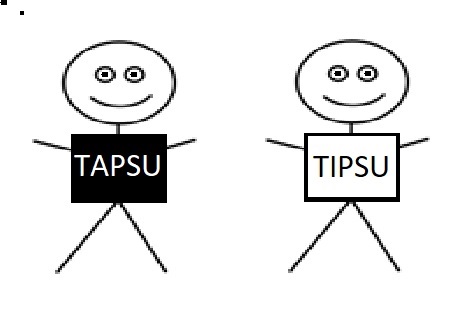* | |  |  | *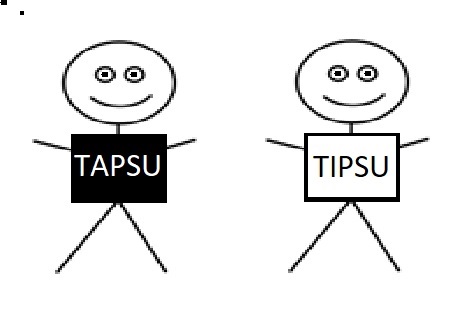* | |  |
|  |  | **□** | **□o** |  |  | **□o** | **□** |  |
|  |  | Just like me | A bit like me |  |  | A bit like me | Just like me |  |
|  |  |  |  |  |  |  |  |  |

|  |  |  | | 3 |  |  | |  |
| --- | --- | --- | --- | --- | --- | --- | --- | --- |
|  | 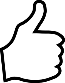  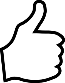  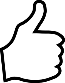 | Dad doesn’t always encourage Tipsu to do physical activity or sports | |  |  | Dad always encourages Tapsu to do physical activity or sports | |  |
|  |  | *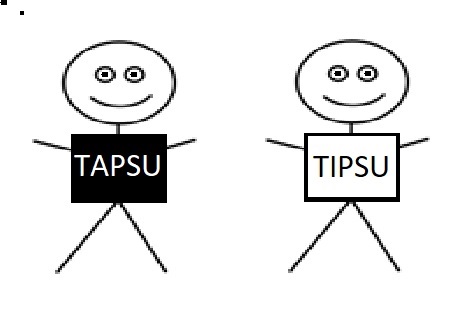* | |  |  | *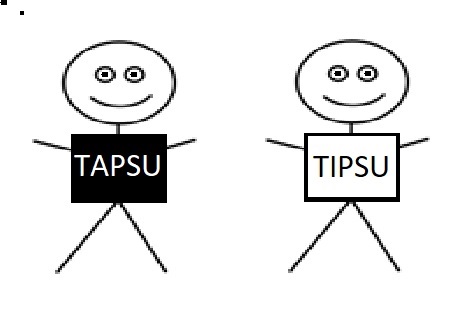* | |  |
|  |  | **□** | **□o** |  |  | **□o** | **□** |  |
|  |  | Just like me | A bit like me |  |  | A bit like me | Just like me |  |
|  |  |  |  |  |  |  |  |  |

|  |  |  | | 4 | |  | |  |
| --- | --- | --- | --- | --- | --- | --- | --- | --- |
|  | 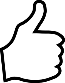  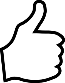  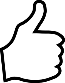  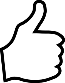 | Dad is always willing to help Tipsu in every way when it comes to physical activity and sports | |  |  | Dad isn’t always willing to help Tapsu in every way when it comes to physical activity and sports | |  |
|  |  | *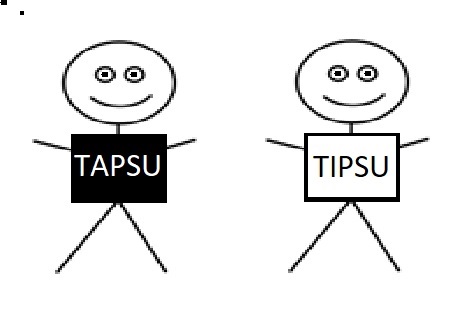* | |  |  | *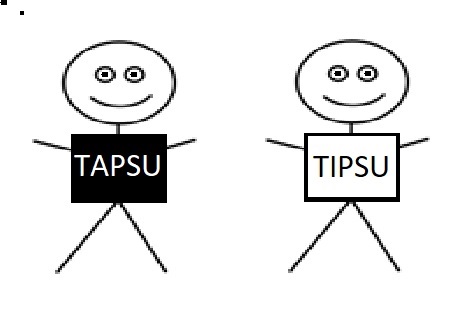* | |  |
|  |  | **□** | **□o** |  |  | **□o** | **□** |  |
|  |  | Just like me | A bit like me |  |  | A bit like me | Just like me |  |
|  |  |  |  |  |  |  |  |  |

|  |  |  | | 5 | |  | |  |
| --- | --- | --- | --- | --- | --- | --- | --- | --- |
|  | 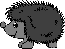 | Dad often decides the style and way that Tipsu should do physical activity (for example, he might say: ‘Not like that, like this!’) | |  |  | Tapsu decides the style and way that Tapsu is physically active | |  |
|  |  | *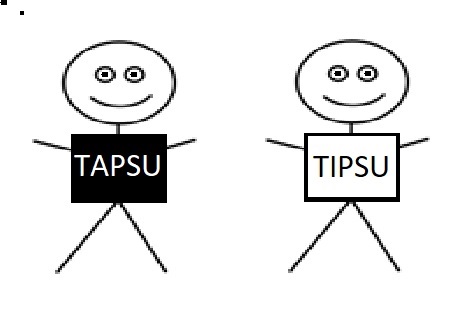* | |  |  | *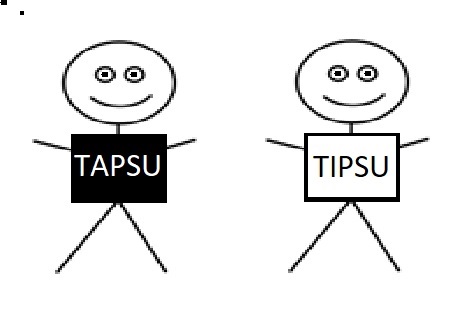* | |  |
|  |  | **□** | **□o** |  |  | **□o** | **□** |  |
|  |  | Just like me | A bit like me |  |  | A bit like me | Just like me |  |
|  |  |  |  |  |  |  |  |  |

|  |  |  | | 6 | |  | |  |
| --- | --- | --- | --- | --- | --- | --- | --- | --- |
|  | 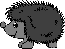  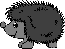 | Tipsu gets to decide how long Tipsu will be physically active for | |  |  | Dad decides how long Tapsu has to do physical activity for (says things like: “Keep going, don’t stop!”) | |  |
|  |  | *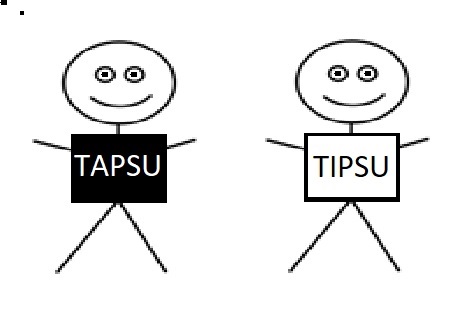* | |  |  | *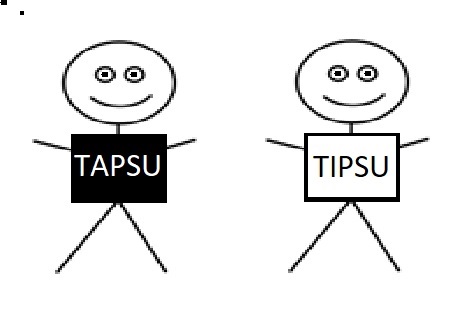* | |  |
|  |  | **□** | **□o** |  |  | **□o** | **□** |  |
|  |  | Just like me | A bit like me |  |  | A bit like me | Just like me |  |
|  |  |  |  |  |  |  |  |  |

|  |  |  | | 7 | |  | |  |
| --- | --- | --- | --- | --- | --- | --- | --- | --- |
|  | 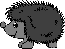  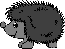  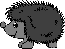 | Dad gives Tipsu strong advice and often does so while Tipsu is physically active (says things like: “Go hard, hard!”, “Focus!”, “Not like that!”) | |  |  | Dad rarely gives Tapsu strong advice while Tapsu is physically active | |  |
|  |  | *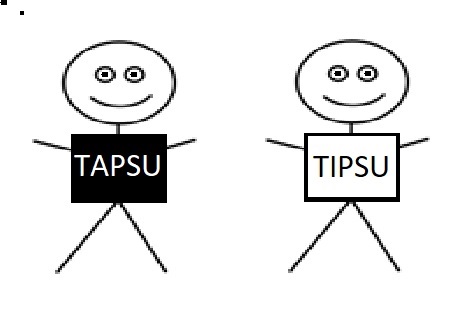* | |  |  | *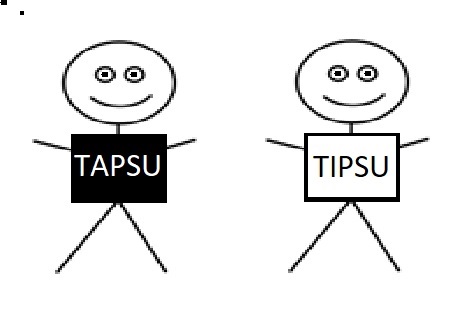* | |  |
|  |  | **□** | **□o** |  |  | **□o** | **□** |  |
|  |  | Just like me | A bit like me |  |  | A bit like me | Just like me |  |
|  |  |  |  |  |  |  |  |  |

|  |  |  | | 8 | |  | |  |
| --- | --- | --- | --- | --- | --- | --- | --- | --- |
|  | 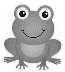 | Tipsu decides the style and way that Tipsu is physically active | |  |  | Mum often decides the style and way that Tapsu should do physical activity (for example, she might say: “Not like that, like this!”) | |  |
|  |  | *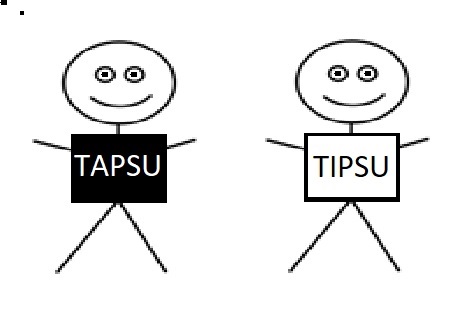* | |  |  | *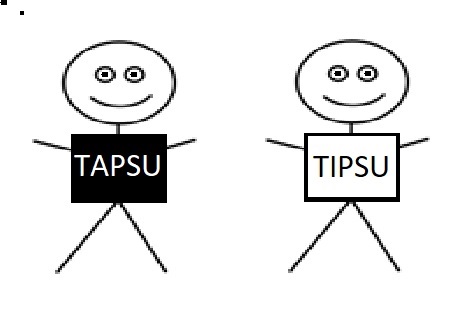* | |  |
|  |  | **□** | **□o** |  |  | **□o** | **□** |  |
|  |  | Just like me | A bit like me |  |  | A bit like me | Just like me |  |
|  |  |  |  |  |  |  |  |  |

|  |  |  | | 9 | |  | |  |
| --- | --- | --- | --- | --- | --- | --- | --- | --- |
|  | 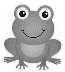  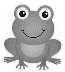 | Mum decides how long Tipsu has to do physical activity for (says things like: “Keep going, don’t stop!”) | |  |  | Tapsu gets to decide how long Tapsu will be physically active for | |  |
|  |  | *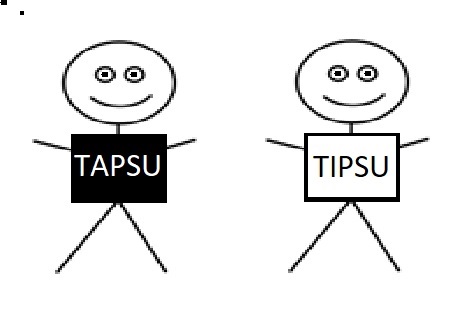* | |  |  | *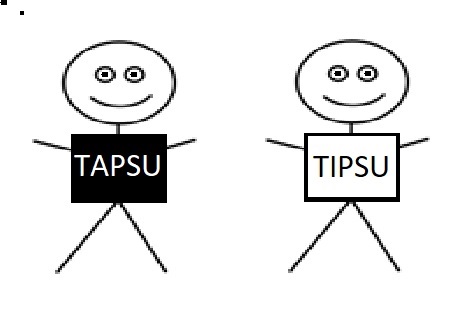* | |  |
|  |  | **□** | **□o** |  |  | **□o** | **□** |  |
|  |  | Just like me | A bit like me |  |  | A bit like me | Just like me |  |
|  |  |  |  |  |  |  |  |  |

|  |  |  | | 10 | |  | |  |
| --- | --- | --- | --- | --- | --- | --- | --- | --- |
|  | 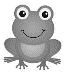  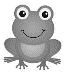  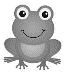 | Mum rarely gives Tipsu strong advice while Tipsu is physically active | |  |  | Mum gives Tapsu strong advice and often does so while Tapsu is physically active (says things like: “Go hard, hard!”, “Focus!”, “Not like that!”) | |  |
|  |  | *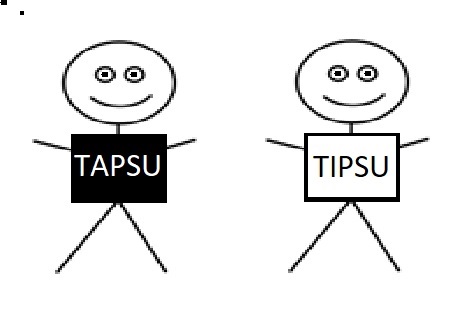* | |  |  | *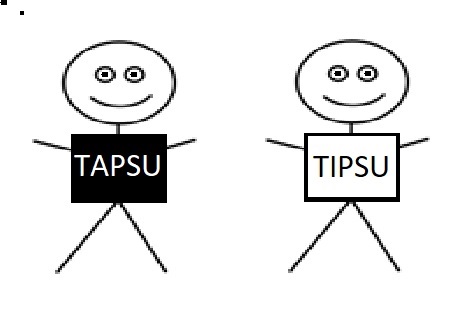* | |  |
|  |  | **□** | **□o** |  |  | **□o** | **□** |  |
|  |  | Just like me | A bit like me |  |  | A bit like me | Just like me |  |
|  |  |  |  |  |  |  |  |  |

|  |  |  | | 11 | |  | |  |
| --- | --- | --- | --- | --- | --- | --- | --- | --- |
|  | 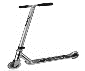 | Dad sometimes ignores it if Tipsu gets tired while being physically active | |  |  | Dad always notices it if Tapsu gets tired while being physically active (asks, for example, “Can you keep going?”) | |  |
|  |  | *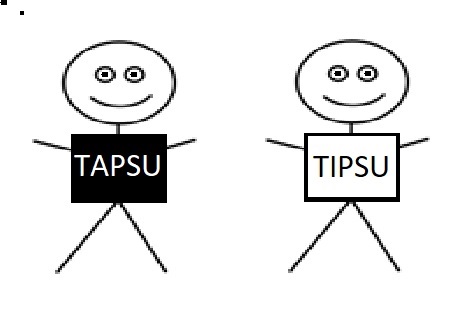* | |  |  | *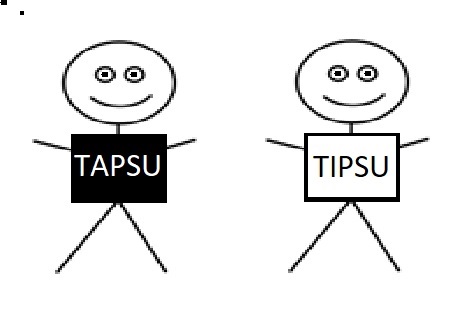* | |  |
|  |  | **□** | **□o** |  |  | **□o** | **□** |  |
|  |  | Just like me | A bit like me |  |  | A bit like me | Just like me |  |
|  |  |  |  |  |  |  |  |  |

|  |  |  | | 12 | |  | |  |
| --- | --- | --- | --- | --- | --- | --- | --- | --- |
|  | 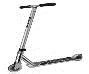  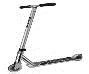 | Dad always listens carefully to what Tipsu has to say about being physically active | |  |  | Dad doesn’t always listen carefully to what Tapsu has to say about being physically active | |  |
|  |  | *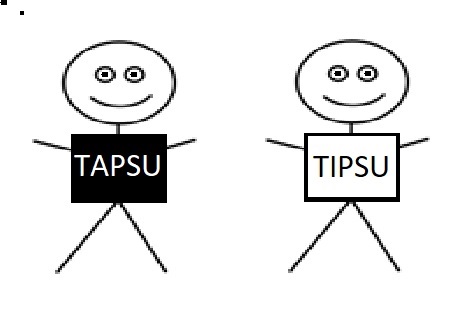* | |  |  | *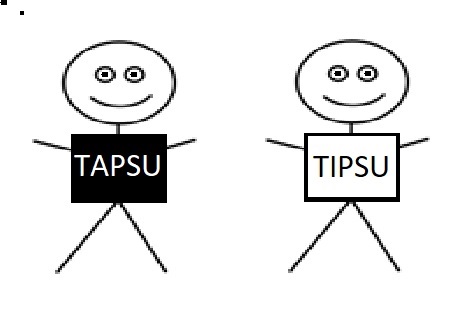* | |  |
|  |  | **□** | **□o** |  |  | **□o** | **□** |  |
|  |  | Just like me | A bit like me |  |  | A bit like me | Just like me |  |
|  |  |  |  |  |  |  |  |  |

|  |  |  | | 13 | |  | |  |
| --- | --- | --- | --- | --- | --- | --- | --- | --- |
|  | 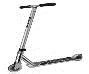  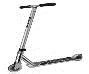  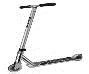 | Dad doesn’t always consider what kind of physical activity Tipsu would or wouldn’t like to do | |  |  | Dad always considers what kind of physical activity Tapsu would or wouldn’t like to do | |  |
|  |  | *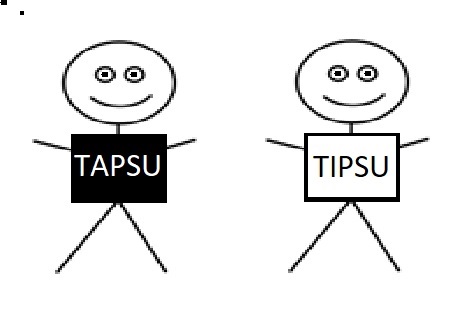* | |  |  | *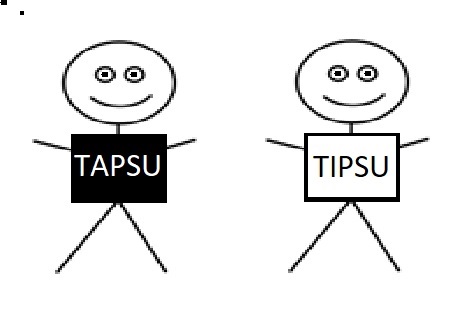* | |  |
|  |  | **□** | **□o** |  |  | **□o** | **□** |  |
|  |  | Just like me | A bit like me |  |  | A bit like me | Just like me |  |
|  |  |  |  |  |  |  |  |  |

|  |  |  | | 14 | |  | |  |
| --- | --- | --- | --- | --- | --- | --- | --- | --- |
|  | 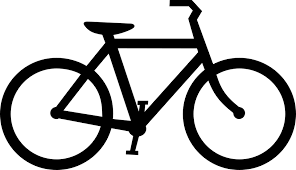 | Mum always notices it if Tipsu gets tired while being physically active (asks, for example, “Can you keep going?”) | |  |  | Mum sometimes ignores it if Tapsu gets tired while being physically active | |  |
|  |  | *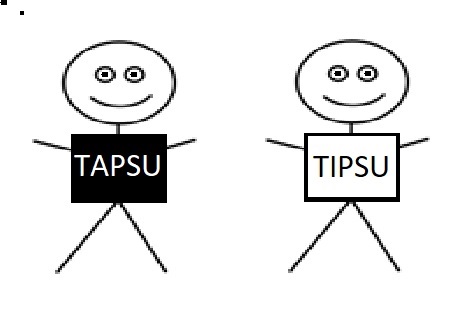* | |  |  | *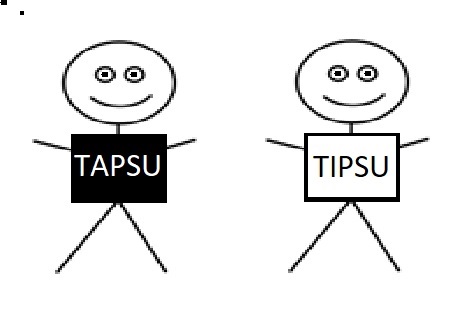* | |  |
|  |  | **□** | **□o** |  |  | **□o** | **□** |  |
|  |  | Just like me | A bit like me |  |  | A bit like me | Just like me |  |
|  |  |  |  |  |  |  |  |  |

|  |  |  | | 15 | |  | |  |
| --- | --- | --- | --- | --- | --- | --- | --- | --- |
|  | 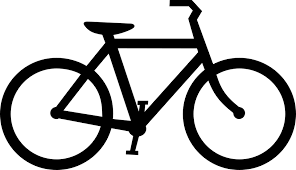  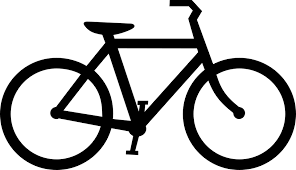 | Mum doesn’t always listen carefully to what Tipsu has to say about being physically active | |  |  | Mum always listens carefully to what Tapsu has to say about being physically active | |  |
|  |  | *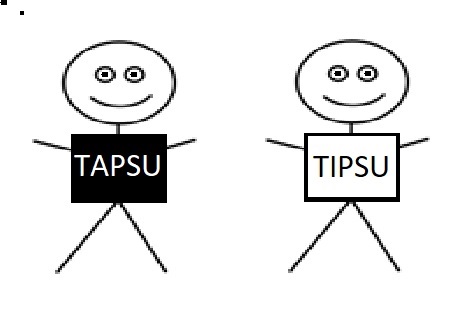* | |  |  | *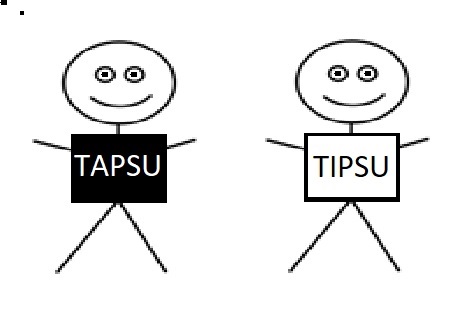* | |  |
|  |  | **□** | **□o** |  |  | **□o** | **□** |  |
|  |  | Just like me | A bit like me |  |  | A bit like me | Just like me |  |
|  |  |  |  |  |  |  |  |  |

|  |  |  | | 16 | |  | |  |
| --- | --- | --- | --- | --- | --- | --- | --- | --- |
|  | 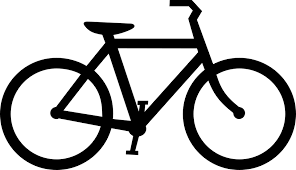  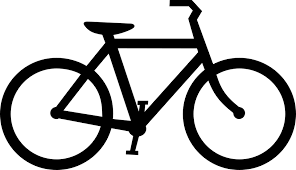  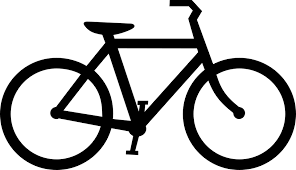 | Mum always considers what kind of physical activity Tipsu would or wouldn’t like to do | |  |  | Mum doesn’t always consider what kind of physical activity Tapsu would or wouldn’t like to do | |  |
|  |  | *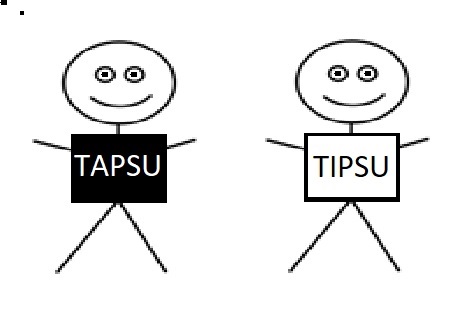* | |  |  | *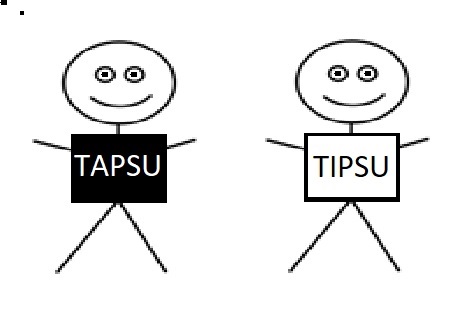* | |  |
|  |  | **□** | **□o** |  |  | **□o** | **□** |  |
|  |  | Just like me | A bit like me |  |  | A bit like me | Just like me |  |
|  |  |  |  |  |  |  |  |  |

|  |  |  | | 17 | |  | |  |
| --- | --- | --- | --- | --- | --- | --- | --- | --- |
|  | 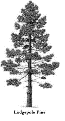 | Tipsu thinks dad is physically active often (for example, dad goes for walks, goes to the gym, or plays ball games) | |  |  | Tapsu thinks dad is not physically active often | |  |
|  |  | *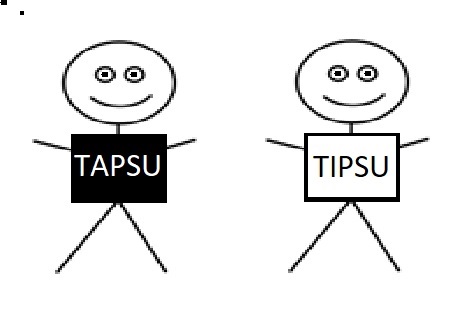* | |  |  | *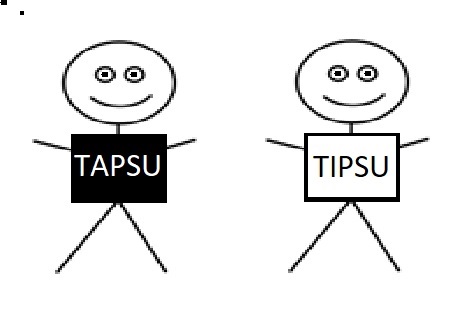* | |  |
|  |  | **□** | **□o** |  |  | **□o** | **□** |  |
|  |  | Just like me | A bit like me |  |  | A bit like me | Just like me |  |
|  |  |  |  |  |  |  |  |  |

|  |  |  | | 18 | |  | |  |
| --- | --- | --- | --- | --- | --- | --- | --- | --- |
|  | 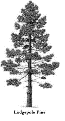  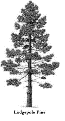 | Tipsu isn’t often physically active with mum and dad | |  |  | Tapsu is often physically active with mum and dad | |  |
|  |  | *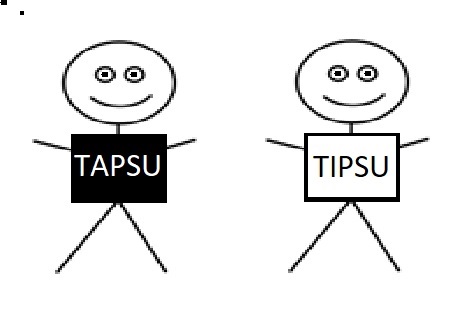* | |  |  | *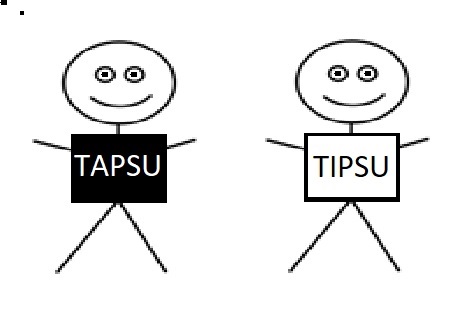* | |  |
|  |  | **□** | **□o** |  |  | **□o** | **□** |  |
|  |  | Just like me | A bit like me |  |  | A bit like me | Just like me |  |
|  |  |  |  |  |  |  |  |  |

|  |  |  | | 19 | |  | |  |
| --- | --- | --- | --- | --- | --- | --- | --- | --- |
|  | 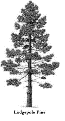  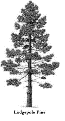  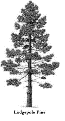 | Parents often drive Tipsu to physical activities or sports practice | |  |  | Parents don’t often drive Tapsu to physical activities or sports practice | |  |
|  |  | *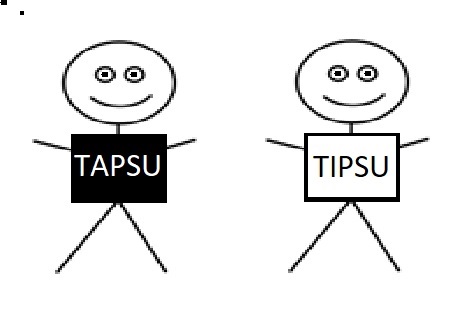* | |  |  | *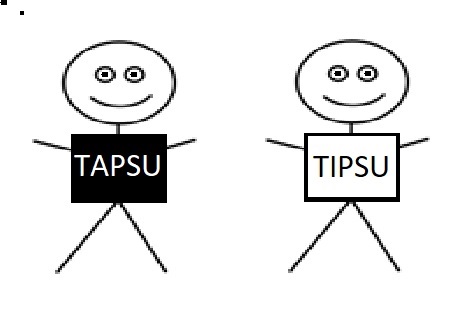* | |  |
|  |  | **□** | **□o** |  |  | **□o** | **□** |  |
|  |  | Just like me | A bit like me |  |  | A bit like me | Just like me |  |
|  |  |  |  |  |  |  |  |  |

|  |  |  | | 20 | |  | |  |
| --- | --- | --- | --- | --- | --- | --- | --- | --- |
|  | 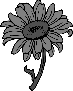 | Tipsu is not physically active with mum | |  |  | Tapsu is physically active with mum (for example, walks, cycles or does sports) | |  |
|  |  | *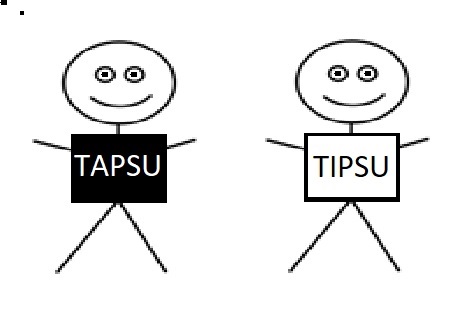* | |  |  | *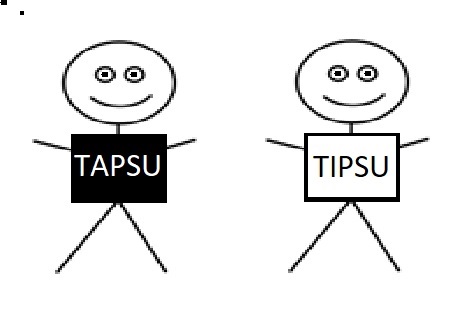* | |  |
|  |  | **□** | **□o** |  |  | **□o** | **□** |  |
|  |  | Just like me | A bit like me |  |  | A bit like me | Just like me |  |
|  |  |  |  |  |  |  |  |  |

|  |  |  | | 21 | |  | |  |
| --- | --- | --- | --- | --- | --- | --- | --- | --- |
|  | 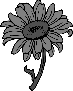  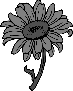 | Tipsu is physically active with dad (for example, walks, cycles or does sports) | |  |  | Tapsu is not physically active with dad | |  |
|  |  | *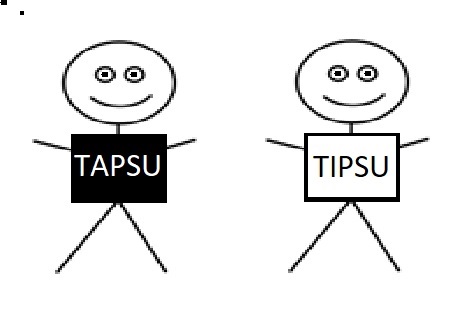* | |  |  | *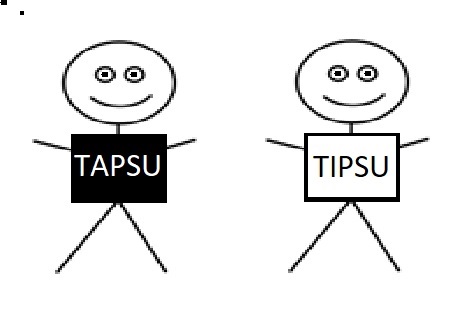* | |  |
|  |  | **□** | **□o** |  |  | **□o** | **□** |  |
|  |  | Just like me | A bit like me |  |  | A bit like me | Just like me |  |
|  |  |  |  |  |  |  |  |  |

|  |  |  | | 22 | |  | |  |
| --- | --- | --- | --- | --- | --- | --- | --- | --- |
|  | 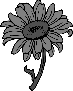  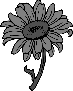  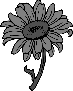 | Tipsu thinks mum is not physically active often | |  |  | Tapsu thinks mum is often physically active (for example, mum goes for walks, goes to the gym, or does sports) | |  |
|  |  | *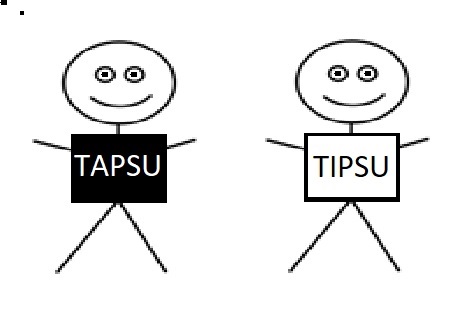* | |  |  | *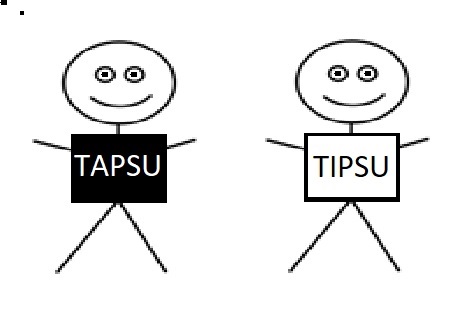* | |  |
|  |  | **□** | **□o** |  |  | **□o** | **□** |  |
|  |  | Just like me | A bit like me |  |  | A bit like me | Just like me |  |
|  |  |  |  |  |  |  |  |  |
