# Supplementary material for: Construct validity and reliability of the physical activity parenting questionnaire for children (PAP-C)
Source: Int J Behav Nutr Phys Act. 2021 May 5;18:61. doi: 10.1186/s12966-021-01128-5 (PMC8097989; doi:10.1186/s12966-021-01128-5)
Supplement: Supplementary file 2 — Additional file 2. Summary of the fit statistics for structure for activity, autonomy support and involvement for the grade levels separately. [file 12966_2021_1128_MOESM2_ESM.docx]

Supplementary material 2

*Summary of the fit statistics for structure for activity, autonomy support and involvement for the grade levels separately*

| Model | χ2 | df | CFI | TLI | RMSEA | SRMR |
| --- | --- | --- | --- | --- | --- | --- |
| Grade 1 |  |  |  |  |  |  |
| First-order, 3-factors, 20 items | 262.547 | 167 | 0.917 | 0.906 | 0.059 | 0.092 |
| Grade 2 |  |  |  |  |  |  |
| First-order, 3-factors, 20 items | 274.539 | 167 | 0.918 | 0.907 | 0.064 | 0.091 |
| Grade 3 |  |  |  |  |  |  |
| First-order, 3-factors, 20 items | 268.458 | 167 | 0.939 | 0.931 | 0.068 | 0.097 |
